# PERSPECTIVES OF GERIATRIC FELLOWS TOWARD SCHOLARLY ACTIVITY

**DOI:** 10.1093/geroni/igad104.1077

**Published:** 2023-12-21

**Authors:** Sadaf Milani, Adeeb Ahmed, Shilpa Rajagopal, Mukaila Raji

**Affiliations:** University of Texas Medical Branch, Galveston, Texas, United States; University of Texas Medical Branch at Galveston, Galveston, Texas, United States; University of Texas Medical Branch, Galveston, Texas, United States; The University of Texas Medical Branch, Galveston, Texas, United States

## Abstract

Previous research conducted nearly two decades ago found that almost half of geriatricians published during their fellowships. About 17% of these geriatricians also indicated a need for further training in scholarly activity. However, it is unclear what barriers and facilitators to participating in scholarly activity geriatric fellows currently face. Our objective was to assess perceived barriers, facilitators, and attitudes toward scholarly activity among current geriatric fellows in ACGME-accredited programs. We conducted a survey of current geriatric fellows from September 2022 to November 2022. An online survey was emailed to geriatric fellows and geriatric program coordinators, when geriatric fellow emails were not publicly available, to assess fellows’ perceived barriers, facilitators, and attitudes toward scholarly activity. We used descriptive statistics to assess geriatric fellows’ perceptions of scholarly activity. Our sample (n=36) was mostly women (61.1%). The most common scholarly activities fellows planned to participate in during fellowship included quality improvement projects (80.6%), case report/series (44.4%), or original research studies (44.4%). The most common perceived major barriers reported were the short duration of fellowship (69.4%) and lack of training to conduct scholarly activity (58.3%). Lastly, about 44.4% of fellows reported being extremely or very enthusiastic and 50% reported being somewhat enthusiastic about participating in research during their fellowship. Most fellows reported plans for at least one type of scholarly activity and reported at least being somewhat enthusiastic about participating in research, however, barriers were identified. A follow-up email survey will be distributed at the completion of fellowship to assess changes in fellow perspectives.

